# Catheter ablation vs antiarrhythmic drugs for persistent atrial fibrillation in hypertrophic cardiomyopathy

**DOI:** 10.1016/j.hroo.2025.09.021

**Published:** 2025-09-26

**Authors:** Zaid Shahrori, Abdalla Rayyan, Sheila Sharma, Loura Sallam, Mauricio Arruda, Judith Mackall, Esseim Sharma

**Affiliations:** 1 Harrington Heart & Vascular Institute, University Hospitals Cleveland Medical Center, Case Western Reserve University, School of Medicine, Cleveland, Ohio; 2 College of Medicine, University of Central Florida, Orlando, Florida; 3 St. George’s University School of Medicine, True Blue, St. George’s, West Indies, Grenada; 4 Faculty of Medicine, University of Alexandria, Alexandria, Egypt

**Keywords:** Atrial fibrillation (AF), Hypertrophic cardiomyopathy (HCM), Persistent atrial fibrillation (PsAF), Catheter ablation (CA), Antiarrhythmic drugs (AADs), Propensity score matching, All-cause mortality, Heart failure exacerbations, Hospitalizations, Electronic health records (EHRs)/TriNetX

**Key Findings**

▪ Catheter ablation (CA) was associated with significantly lower 12-month all-cause mortality compared with antiarrhythmic drug (AAD) therapy in patients with hypertrophic cardiomyopathy (HCM) and persistent atrial fibrillation (PsAF).
▪ CA significantly reduced heart failure exacerbations requiring intravenous diuretics, hospitalizations, and continued AAD use vs AAD therapy alone.
▪ Kaplan-Meier survival analysis demonstrated higher survival probabilities in the CA group (98.8%) than in the AAD group (96.5%), with a hazard ratio of 0.348 (*P* < .0001).
▪ Findings suggest that CA-based rhythm control may offer superior clinical outcomes compared with AAD therapy in patients with HCM and PsAF, though prospective studies are needed to validate these results.

## Introduction

Atrial fibrillation (AF) is the most common arrhythmia in patients with hypertrophic cardiomyopathy (HCM), affecting 20%–25% of patients, and is associated with an increased risk of heart failure, stroke, and mortality.^1^ Managing AF in HCM is uniquely challenging because of myocardial remodeling, autonomic dysfunction, and inflammatory responses, which predispose these patients to arrhythmias with many progressing to persistent AF (PsAF), which is poorly tolerated.^1^ Although catheter ablation (CA) has been shown to be more effective than antiarrhythmic drugs (AADs) for patients with PsAF in maintaining sinus rhythm and significantly reducing AF burden and symptoms, it remains unclear whether this advantage extends to patients with HCM and translates into clinical outcomes.^2^

This retrospective cohort study compares 12-month outcomes of CA and AADs in patients with AF and HCM. Using the TriNetX platform, a multicenter federated health research network aggregating data of >250 million patients’ electronic health records, we analyzed 1827 patients with HCM (both obstructive and nonobstructive) and PsAF treated with CA and 3641 patients with HCM and PsAF treated solely with AADs.^3^ A 90-day postablation blanking period was used to minimize confounding by acute procedural effects. Adjusted odds ratios with 95% confidence intervals were calculated. Index events were the date of ablation for the CA group and the date of AADs initiation for the AAD group. The research reported in this article adhered to the principles of the Declaration of Helsinki. This retrospective study used de-identified electronic health record data from the TriNetX network and therefore did not require individual patient consent.

Kaplan-Meier curves created using log-rank tests assessed survival differences. Statistical significance was set at a *P* value <0.05. Propensity score matching (greedy nearest-neighbor algorithm, caliper = 0.1 pooled standard deviations) resulted in 2 demographically and clinically balanced cohorts of 1694 patients each.

Prior to matching, the CA cohort was younger (62.9 years vs 66.9 years) and had a higher proportion of men (68.9% vs 57.8%). After propensity score matching, demographic and clinical variables were well-matched between the 2 cohorts (Table 1).

**Table 1:** Baseline characteristics and clinical outcomes after PSM

Baseline characteristic	Before PSM			After PSM		
	HCM and CA (n = 1827)	HCM and AAD (n = 3641)	*P*	HCM and CA (n = 1694)	HCM and AAD (n = 1694)	*P*
Demographic characteristics						
Age (y)	62.9 ± 10.9	66.9 ± 12.4	<.001	63.6 ± 10.5	64.0 ± 12.7	.329
Male sex	68.90	57.80	<.001	67.60	67.20	.798
Race and ethnicity						
Asian	2.6	2.9	.553	2.6	2.1	.365
Black	11.8	16.1	<.001	12.5	11.5	.397
Hispanic or Latino	3.0	3.3	.611	3.0	4.1	.076
White	80.3	75.2	<.001	79.8	81.3	.259
Comorbidities						
Hypertensive diseases	77.2	73.5	.003	77.4	77.9	.741
Ischemic heart disease	51.9	50.8	.449	53.0	53.7	.654
Heart failure	50.5	50.4	.972	51.7	51.5	.918
Cerebrovascular disease	15.1	21.0	<.001	16.3	16.0	.815
Obstructive sleep apnea	37.1	23.9	<.001	34.5	34.4	.942
Diabetes	26.0	28.9	.025	27.2	26.9	.816
Cocaine-related disorders	1.7	1.6	.743	1.7	1.9	.698
Alcohol-related disorders	8.0	7.1	.26	7.9	9.6	.088
Tobacco use	7.5	7.3	.829	7.5	8.8	.167
Miscellaneous						
LV ejection fraction (%)	53.9 ± 13.9	52.6 ± 16.8	.088	53.8 ± 13.8	52.6 ± 16.7	.156
Body mass index (kg/m^2^)	31.6 ± 6.6	30.9 ± 7.5	.003	31.4 ± 6.5	31.7 ± 7.7	.184

Outcome after PSM	HCM and CA (n = 1694)	HCM and AAD (n = 1694)	OR (95% CI)	*P*
Heart failure	6.4	11.5	0.52 (0.41–0.67)	<.0001
Hospitalizations	31.2	38.8	0.72 (0.62–0.83)	<.0001
AAD	30.8	42.9	0.59 (0.51–0.68)	<.0001
Cardioversion	11.9	6.4	1.99 (1.56–2.54)	<.0001
CVA	3.8	4.7	0.79 (0.57–1.11)	.173
All-cause mortality	1.2	3.4	0.35 (0.21–0.58)	<.0001

This table presents baseline demographic and clinical characteristics of patients with HCM and PsAF treated with CA vs AAD, both before and after 1:1 PSM. Postmatching comparisons of 12-mo clinical outcomes are also included. Comparisons are reported with *P* values and adjusted ORs with 95% CIs. Count (percentage) are shown for categorical variables.

AAD = antiarrhythmic drug; CA = catheter ablation; CI = confidence interval; CVA = cerebrovascular accident; HCM = hypertrophic cardiomyopathy; LV = left ventricular; OR = odds ratio; PsAF = persistent atrial fibrillation; PSM = propensity score matching.

The CA cohort had a significantly lower all-cause mortality rate (1.2% vs 3.4%), and Kaplan-Meier analysis showed a 12-month survival probability of 98.8% in the CA cohort vs 96.5% in the AAD cohort, with a hazard ratio of 0.348 (log-rank, *P* < .0001). CA was also associated with significantly lower rates of heart failure exacerbations requiring intravenous diuretics (6.4% vs 11.5%), hospitalizations (31.2% vs 38.8%), and continued AAD use (30.8% vs 42.9%) (Table 1).

These findings suggest that a CA-based strategy may be associated with significant benefits when compared with AAD therapy alone in propensity score–matched patients with HCM and PsAF, demonstrating significant reductions in all-cause mortality and hospitalizations. 1 potential explanation for these findings is superior rhythm control in the CA group. Rhythm control was abandoned in many patients initially treated with AADs, with only 42.9% of them remaining on AADs by the end of the study. This suggests that many patients in the AAD group transitioned to a rate control strategy, whereas in the ablation group, there was likely a greater effort to maintain sinus rhythm, contributing to the increased rates of cardioversion observed. The improvements in outcomes seen in this study align with improvements observed in patients with heart failure—both with reduced and preserved ejection fraction—who undergo CA.^4^ Moreover, the reduced reliance on AADs in the CA cohort may be clinically meaningful, as long-term AADs use has been associated with adverse cardiovascular outcomes and increased mortality.^5^

A key limitation of this study is the absence of continuous rhythm monitoring, which precludes definitive assessment of arrhythmia recurrence and complicates attribution of benefits solely to rhythm control. Additionally, the retrospective design and reliance on electronic health record data introduce potential biases, such as selection bias and miscoding. Lastly, unmeasured confounders may contribute to the observed differences between cohorts despite propensity score matching.

In conclusion, when compared with AADs use alone, CA in patients with HCM and PsAF is associated with reductions in mortality, hospitalizations, and AADs dependency in a propensity score–matched cohort. These findings suggest that despite the challenging substrate and lower historical success rates, CA may favorably affect patient outcomes. Prospective studies are needed to confirm these results and refine clinical guidelines.

## Disclosures

The authors have no conflicts of interest to disclose.
